# Management of Immune‐Mediated Thrombotic Thrombocytopenic Purpura Without Plasma Exchange in a Jehovah’s Witness: A Case Report

**DOI:** 10.1155/crh/2753797

**Published:** 2026-11-09

**Authors:** Carina Wong, Rakesh Mehta

**Affiliations:** ^1^ School of Medicine Indiana University 1030 W. Michigan St., 2nd Floor Indianapolis 46202 Indiana USA indiana.edu

**Keywords:** case report, immune-mediated thrombotic thrombocytopenic purpura, intravenous immunoglobulin, Jehovah’s Witness, rituximab

## Abstract

Immune‐mediated thrombotic thrombocytopenic purpura (iTTP) is a life‐threatening thrombotic microangiopathy caused by severe ADAMTS13 deficiency and is traditionally treated with urgent therapeutic plasma exchange. Management becomes particularly challenging in patients who decline blood products. We report the case of a previously healthy woman with severe iTTP, with a platelet nadir of 6 × 10^9^/L, ADAMTS13 activity < 5%, and an ADAMTS13 inhibitor titer of 2 Bethesda units, who declined plasma exchange because of her religious beliefs as a member of Jehovah’s Witnesses. She was treated with high‐dose corticosteroids, rituximab, and adjunctive intravenous immunoglobulin (IVIG). Platelet count normalized by hospital Day 9 with progressive resolution of hemolysis, and at 6‐month follow‐up the patient remained in durable clinical and biochemical remission with normalization of ADAMTS13 activity. This case highlights the potential adjunctive role of IVIG within an immune‐directed treatment strategy for iTTP when plasma exchange is not feasible.

## 1. Introduction

Immune‐mediated thrombotic thrombocytopenic purpura (iTTP) is characterized by a microangiopathic hemolytic anemia, thrombocytopenia, and severe deficiency of ADAMTS13 activity due to inhibitory autoantibodies [1]. The current standard of care includes urgent therapeutic plasma exchange in combination with immunosuppressive therapy [2]. Management of iTTP in patients who are members of the Jehovah’s Witness religious community needs to be individualized due to their beliefs regarding the use of blood products, including plasma exchange. Plasma exchange–free treatment strategies for iTTP remain poorly characterized, with the available evidence largely limited to small observational studies and case reports.

## 2. Case

The patient is a 45‐year‐old female who noticed a headache the evening prior to her hospitalization. The following morning, she developed abdominal pain, nausea, vomiting, and subsequently went to the emergency department. Initial laboratory evaluation at an outside hospital demonstrated a hemoglobin of 11.4 g/dL and a platelet count of 6 × 10^9^/L, which represented the platelet nadir during her hospitalization. Hemolysis markers were notable for elevated lactate dehydrogenase of 957 U/L and total bilirubin of 4.7 mg/dL with a direct fraction of 0.5 mg/dL, and a decreased haptoglobin of 10 mg/dL. Her peripheral smear revealed numerous schistocytes, consistent with a microangiopathic hemolytic anemia. ADAMTS13 activity returned as undetectable (< 5%), and an ADAMTS13 inhibitor was detected at 2 Bethesda units, confirming iTTP. Renal function was preserved, and there were no focal neurologic deficits at presentation.

As a member of Jehovah’s Witnesses, the patient declined the transfusion of blood products, including plasma exchange and fresh frozen plasma. The use of intravenous immunoglobulin (IVIG) was discussed in detail with the patient and her family, with additional consultation with a church patient advocate regarding the derivation of IVIG. After these discussions, she consented to its use in her treatment regimen.

Therapy was directed toward rapid suppression of the underlying autoimmune process. Treatment was initiated on hospital Day 1 with high‐dose intravenous methylprednisolone (1000 mg daily for 3 days), followed by oral prednisone 80 mg daily with a gradual taper. Rituximab 375 mg/m^2^ weekly was initiated on hospital Day 2. The platelet count initially increased to 18 × 10^9^/L following corticosteroid initiation but subsequently declined to 8 × 10^9^/L on hospital Day 2. Given the inadequate hematologic response, adjunctive IVIG was initiated on hospital Day 3 at a dose of 1 g/kg daily for 2 days. The patient ultimately received a total of four rituximab doses, including a second dose on hospital Day 9 and two additional doses in the outpatient setting.

Following initiation of combination therapy, the platelet count demonstrated a steady increase reaching 153 × 10^9^/L by hospital Day 9, the day of discharge. Hemoglobin reached a nadir of 5.8 g/dL on hospital Day 4 and subsequently recovered more gradually following discharge. Lactate dehydrogenase remained mildly elevated at 437 U/L at discharge but continued to improve thereafter. At 1‐week follow‐up, the platelet count was 242 × 10^9^/L, hemoglobin had increased to 8.9 g/dL, haptoglobin had normalized to 73 mg/dL, and lactate dehydrogenase had decreased to 258 U/L, consistent with ongoing resolution of hemolysis. ADAMTS13 activity increased from < 5% at discharge to 34% at 1‐week follow‐up and 71% at 6 months. At 12‐month follow‐up, the patient remained in remission, with platelet count and hemoglobin within normal limits and ADAMTS13 activity greater than 80%. The patient’s hematologic and ADAMTS13 trends during hospitalization and follow‐up are summarized in Table 1.

**TABLE 1:** Hematologic and ADAMTS13 trends during hospitalization and follow‐up.

Time	Platelets (× 10^9^/L)	Hemoglobin (g/dL)	LDH (U/L)	Haptoglobin (mg/dL)	ADAMTS13 activity	ADAMTS13 inhibitor (BU)
Initial presentation (OSH)	6	11.4	957	10	< 5%	—
Hospital Day 1	18	10.4	—	—	—	2.0
Hospital Day 2‐rituximab initiated	8	8.6	—	—	—	—
Hospital Day 3‐IVIG initiated	10	7.2	695	—	—	—
Hospital Day 4	21	5.8	604	—	—	—
Hospital Day 5	34	6.8	625	—	—	—
Hospital Day 6	47	7.5	855	—	—	—
Hospital Day 7	93	7.2	543	—	—	—
Hospital Day 8	127	6.5	449	—	—	—
Hospital Day 9/Discharge	153	6.5	437	—	< 5%	—
1‐week follow‐up	242	8.9	258	73	34%	—
6‐month follow‐up	227	11.8	—	86	71%	—
12‐month follow‐up	211	12.5	250	114	91%	—

*Note:* OSH, outside hospital; LDH, lactate dehydrogenase; Dashes indicate values not available or not measured at the specified time point.

Abbreviation: BU, Bethesda units.

Caplacizumab was considered at presentation and was not precluded by the patient’s religious beliefs; however, due to logistical and accessibility limitations at the time of admission, it could not be promptly initiated. IVIG was more readily available and was therefore selected as adjunctive therapy. Caplacizumab remained an option for escalation in the event of continued or worsening disease; however, the patient subsequently demonstrated progressive hematologic recovery without the need for escalation of therapy.

## 3. Discussion

Corticosteroids remain part of frontline therapy for iTTP because of their immunosuppressive effects [2], including reduction of autoantibody production and modulation of reticuloendothelial system activity. Per International Society on Thrombosis and Haemostasis (ISTH) guidelines, rituximab carries a Level‐2 recommendation in the management of iTTP [2]. Its therapeutic effect is mediated through B‐cell depletion and suppression of new antibody production [3]. However, rituximab does not directly eliminate preexisting circulating autoantibodies. Most published cases describing management of iTTP in patients of Jehovah’s Witness religious community have utilized corticosteroids and rituximab, with caplacizumab incorporated in eligible cases [4].

Data regarding adjunctive IVIG use in iTTP remains limited. One proposed mechanism of IVIG in autoimmune regulation is competitive binding to FcRn receptors, thereby promoting degradation of circulating immunoglobulins, including pathogenic autoantibodies [5]. IVIG may also exert immunomodulatory effects through anti‐idiotypic antibodies capable of neutralizing pathogenic autoantibodies [5].

A prior case report described successful treatment with corticosteroids and IVIG in a pediatric patient with iTTP, while another report involving a member of Jehovah’s Witnesses required subsequent escalation to cryosupernatant replacement therapy despite adjunctive IVIG [6, 7]. Notably, the authors of that report acknowledged that delays in initiation of therapeutic plasma exchange made it difficult to determine the relative contribution of plasma exchange versus immunosuppressive therapy to the patient’s eventual recovery.

In our patient, alternative agents including caplacizumab, vincristine, and bortezomib were considered initially and in the event of inadequate responses. Due to logistical and accessibility limitations, IVIG was more readily available and was therefore initiated as adjunctive therapy. Fortunately, the patient demonstrated progressive hematologic recovery without the need for escalation of therapy. IVIG was incorporated into a broader frontline immunosuppressive strategy consisting of corticosteroids and rituximab, resulting in durable clinical and biochemical remission without plasma exchange or cryoprecipitate‐based therapy. Although the precise contribution of IVIG remains unclear, its immunomodulatory effects may provide adjunctive benefit in selected patients when plasma exchange is not feasible, particularly when access to other therapies such as caplacizumab is limited. Further studies are needed to better define the potential role of IVIG in plasma exchange–free management of iTTP.

## Funding

The authors received no specific funding for this work.

## Disclosure

All authors have read and approved the final version of the manuscript. Rakesh Mehta had full access to all of the data in this study and takes complete responsibility for the integrity of the data and the accuracy of the data analysis.

## Ethics Statement

Informed consent was obtained from the patient for publication of this case report. All details have been anonymized to ensure privacy.

## Conflicts of Interest

The authors declare no conflicts of interest.

## Supporting Information

Additional supporting information can be found online in the Supporting Information section.

## Supporting information


**Supporting Information** revisedCARE‐checklist‐English‐2013%20Wong.

## Data Availability

All data relevant to this case report are included within the article. Additional details are available from the corresponding author upon reasonable request.
