# Supplementary material for: Management of Immune‐Mediated Thrombotic Thrombocytopenic Purpura Without Plasma Exchange in a Jehovah’s Witness: A Case Report
Source: Case Rep Hematol. 2026 Nov 9;2026:2753797. doi: 10.1155/crh/2753797 (PMC13746378; doi:10.1155/crh/2753797)
Supplement: Supplementary file 1 [file CRH-2026-2753797-s001.pdf]

CARE Checklist of information to include when writing a case report

CARE case report guidelines

(cc) BY-NC-ND

Topic	Item	Checklist item description	Reported on Line
Title	1	The diagnosis or intervention of primary focus followed by the words "case report"	Lines 2-3
Key Words	2	2 to 5 key words that identify diagnoses or interventions in this case report, including "case report"	Lines 29-30
Abstract (no references)	3a	Introduction: What is unique about this case and what does it add to the scientific literature?	Lines 17-28
	3b	Main symptoms and/or important clinical findings	Lines 20-23
	3c	The main diagnoses, therapeutic interventions, and outcomes	Lines 20-27
	3d	Conclusion—What is the main "take-away" lesson(s) from this case?	Lines 27-28
Introduction	4	One or two paragraphs summarizing why this case is unique (**may include references**)	Lines 32-39
Patient Information	5a	De-identified patient specific information.	Lines 41-51
	5b	Primary concerns and symptoms of the patient.	Lines 41-43
	5c	Medical, family, and psycho-social history including relevant genetic information	Lines 41,52-56
	5d	Relevant past interventions with outcomes	N/A new presentation
Clinical Findings	6	Describe significant physical examination (PE) and important clinical findings.	Lines 48-51
Timeline	7	Historical and current information from this episode of care organized as a timeline	Lines 41-82, table 1
Diagnostic Assessment	8a	Diagnostic testing (such as PE, laboratory testing, imaging, surveys).	Lines 44-51, table 1
	8b	Diagnostic challenges (such as access to testing, financial, or cultural)	Lines 52-56,77-82
	8c	Diagnosis (including other diagnoses considered)	Lines 48-51
	8d	Prognosis (such as staging in oncology) where applicable	N/A
Therapeutic Intervention	9a	Types of therapeutic intervention (such as pharmacologic, surgical, preventive, self-care)	Lines 57-65,77-82
	9b	Administration of therapeutic intervention (such as dosage, strength, duration)	Lines 59-65
	9c	Changes in therapeutic intervention (with rationale)	Lines 61-65,77-82
Follow-up and Outcomes	10a	Clinician and patient-assessed outcomes (if available)	Lines 66-76
	10b	Important follow-up diagnostic and other test results	Lines 67-76; table 1
	10c	Intervention adherence and tolerability (How was this assessed?)	Lines 57-65
	10d	Adverse and unanticipated events	N/A
Discussion	11a	A scientific discussion of the strengths AND limitations associated with this case report	Lines 104-114
	11b	Discussion of the relevant medical literature **with references**.	Lines 85-103
	11c	The scientific rationale for any conclusions (including assessment of possible causes)	Lines 93-114
	11d	The primary "take-away" lessons of this case report (without references) in a one paragraph conclusion	Lines 110-114
Patient Perspective	12	The patient should share their perspective in one to two paragraphs on the treatment(s) they received	Not provided
Informed Consent	13	Did the patient give informed consent? Please provide if requested	Yes ☑ No ☐
